# Data-Driven Complete Basis Set Limit Estimates from a Minimal Auxiliary Basis

**DOI:** 10.1021/acs.jctc.6c01004

**Published:** 2026-10-19

**Authors:** Nicolas Grimblat, Gabriel Klassen, Guido Falk von Rudorff

**Affiliations:** † Institut für Chemie, 9178 Universität Kassel, Heinrich-Plett-Straße 40, Kassel 34132, Germany; ‡ Center for Interdisciplinary Nanostructure Science and Technology (CINSaT), Heinrich-Plett-Straße 40, Kassel 34132, Germany

## Abstract

Quantum chemistry calculations are often performed using atom-centered basis sets, which are chosen to balance accuracy and cost. While they are systematically improvable, the total energy converges slowly with basis set size toward the complete basis set (CBS) limit. Common extrapolation methods require several intermediate-quality calculations to afford an estimate of the CBS energy. We propose a minimal complementary auxiliary basis set (CABS) baseline to estimate the CBS energy from a single quantum chemistry calculation in a minimal basis set via kernel ridge regression (KRR), which is more efficient than both direct and Δ-machine learning and 40× faster than triple-ζ single-point calculations. The spatial and alchemical gradients from minimal basis and minimal CABS are more accurate than double-ζ (DZ) calculations. We show that KRR on standard molecular representations can be improved by approximating atom-wise local kernels using Chebyshev polynomials, which allows us to train KRR models 10×–50× times faster on moderate compute resources, thereby enabling a data-driven approach toward the mean-field CBS, combining physical baselines capturing leading-order effects with data-efficient machine learning models.

## Introduction

Accurate prediction of molecular energies across broad chemical space still critically depends on controlling basis-set incompleteness error (BSIE). In practice, many workflows require balancing computational cost against proximity to the complete basis set (CBS) limit. Correlation-consistent basis sets (e.g., cc-pVXZ, *X* = *D*, *T*, *Q*, 5, 6) were designed to recover correlation energy (*E*_corr_) in a near-systematic fashion, enabling extrapolation to the CBS limit,^1^ e.g., with inverse–power laws of the form

1 $$E_{\mathrm{corr}}^{X}=E_{\mathrm{corr}}^{\mathrm{CBS}}+AX^{-3}$$

where *X* is the cardinal number.^2^ This concept has been widely adopted and generalized for other systems and methods.^3^ Systematic analyses of Hartree–Fock and correlated convergence have shown regular asymptotic trends as well as method-dependent deviations.^2,4^ For this reason, the choice of extrapolation formula and cardinal-pair selection remains critical in practical workflows,^5–7^ but their reliability can degrade when the selected basis-set pair is not fully within the asymptotic convergence regime. In general, the total energy at the cardinal number *X* can be written as

2 $$E\left(X\right)=E_{\mathrm{CBS}}+Af\left(X\right)$$

where *E*_CBS_ is the CBS limit, *A* is a system-dependent coefficient, and *f*(*X*) describes the decay of the BSIE. Different such decay functions have been proposed and evaluated both for correlated methods^8–13^ and for mean-field method extrapolation,^4,9,14–22^ where practical results often suggest a different decay function than theoretical motivation suggests.^5^ Using a linear combination of two calculations with different basis sets at cardinal numbers *X* and *Y*, elimination of *A* yields a two-point extrapolation framework, e.g., for correlation energies at the CBS $\left(E_{\mathrm{corr}}^{\mathrm{CBS}}\right)$

3 $$E_{\mathrm{corr}}^{\mathrm{CBS}}=\frac{X^{\beta }E_{\mathrm{corr}}\left(X\right)-Y^{\beta }E_{\mathrm{corr}}\left(Y\right)}{X^{\beta }-Y^{\beta }}$$

where *f*(*X*) = *X* ^–β^ controls the rate of the asymptotic convergence. While β = 3 is regularly assumed in the asymptotic regime, several studies have found deviations for lower cardinal numbers.^9,23^ Recent benchmarks further emphasize that these exponents are not universal constants, but depend on both the method and the basis set family, such that reoptimization for each pair can significantly improve accuracy across the chemical space.^6,7^

For Hartree–Fock (HF) energies, convergence with respect to basis set size is typically faster and better described by an exponential-square-root form,^17,24^

4 $$E_{\mathrm{HF}}\left(X\right)=E_{\mathrm{HF}}^{\mathrm{CBS}}+A\exp\left(-\alpha \sqrt{X}\right)$$

where α governs residual HF BSIE decay. In practice α and β are effective parameters that may be optimized empirically for a given basis-set.

By contrast, F12 methods^25^ are explicitly correlated and instead address the slow convergence at its origin, the electron–electron cusp. By incorporating interelectronic-distance dependence directly, F12 methods accelerate convergence substantially relative to conventional expansions and can recover near-CBS energies with substantially smaller basis sets,^2,7,26–29^ mostly with two cardinal numbers less,^27,30^ owing to improved *X* ^–7^ scaling.^31^ Modern F12 implementations rely on auxiliary basis sets, resolution-of-the-identity approximations (RI), and optimized short-range correlation factors^32–37^ F12 requires electron correlation to be included in the underlying quantum chemistry method, so it is not directly applicable to Hartree–Fock, but can be extended to address it using the complementary auxiliary basis set (CABS) singles term^38^ (denoted CABSs in the following) which will be used to build a physics-informed but data-driven correction in this work. The CABS forms a larger basis function space where the singles correction is the energy relaxation of a regular Hartee-Fock calculation which is not CABS-aware when the additional CABS basis functions are added perturbatively.^39^ The CABS space is commonly constructed using optimized auxiliary basis sets (OptRI),^40^ which is similar in spirit to this work, since it optimizes the CABS basis to reproduce MP2-F12 energy contributions. While we reduce the basis even further, CABS/OptRI residuals have large signed errors^41^ which benefit from an atomic correction, but still can form a valid starting point for Coupled-Cluster methods.^42^ Other formalisms pursue variants which can be applied to wave function methods.^43^ For density functional theory (DFT), some employ a single-step correction from a dual basis^44^ or a perturbative approach,^45^ both avoiding diagonalization in the large basis but at a higher cost and larger errors than our approach.^45^

Modern machine learning (ML) approaches regularly adopt an atomic decomposition of total energies, where each atomic contribution depends on the local environment both for direct learning of the total energy^46–53^ and for only considering the CBS correction.^54–58^ For the CBS limit of correlated methods, this typically involves learning extrapolated energies.^59^ Alternative approaches focus on proposing new interactions,^60^ analyzing the emergent interactions^61^ yielding a correlated complete basis set result, or making basis sets system-dependent.^62,63^

The hierarchical structure of the problem naturally supports detrending: a physics-based baseline energy model (e.g., low-cost DFT, or approximate CCSD­(T)/finite-basis result) is decomposed into atomic or local contributions, and the ML model learns a residual correction, commonly referred to as Δ-ML.^64^ This exploits that the learning curve (i.e., the expected error as a function of training data points *N*) scales with the standard deviation of the labels σ_*y*_: reducing the standard deviation by only considering the difference to a baseline shifts the learning curve, effectively reducing the total number of data points needed for learning the same quantity at the added cost of the baseline evaluation. The generalization thereof to cases with an exact hierarchy of basis sets and levels of theory is multifidelity learning^65–67^ which builds linear combinations of individual submodels similar to an ensemble estimator.

Reducing the basis set incompleteness error is relevant^68–70^ for many methods relying on or calculating forces and other gradients such as geometry optimization and molecular dynamics,^71,72^ differentiable chemistry,^73–76^ quantum alchemy,^77–80^ response functions, and perturbation theory.^81–83^

In the long history of approximations in quantum chemistry, elements naturally have been treated as distinct entities, for example, in the design of pseudopotentials or basis functions. This is a particular issue for mixed response functions, that is, where geometric changes are coupled with electronic changes.^84^ Since the Hellmann–Feynman theorem, which conceptually offers a particularly convenient calculation of electronic gradients, only holds if the basis set does not change in the direction of that derivative, Pulay correction terms have to be included for incomplete basis sets. In this work, we focus on Hartree–Fock CBS, since it is the dominant physical component to corrections of higher level methods, and published databases offer a large amount of data that allow for a more principled analysis of this method, as outlined in Figure 1.

**Figure 1:**
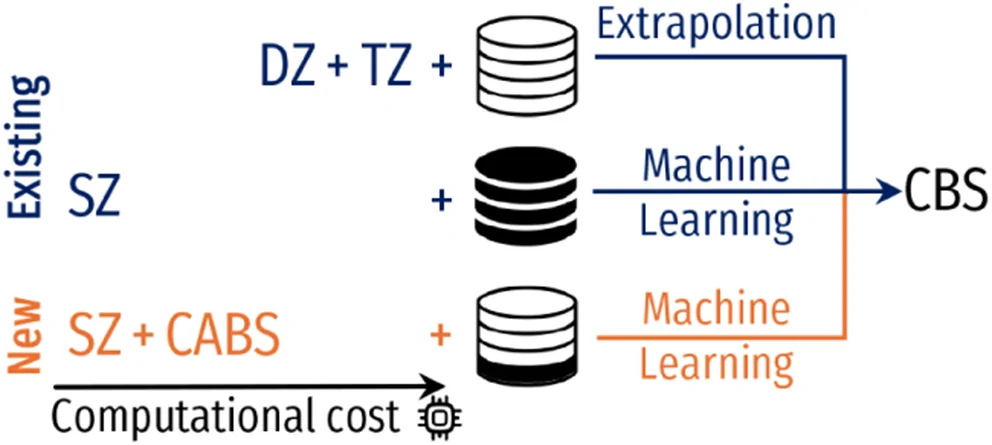
Comparison of workflows toward the estimation of the complete basis set limit. Top: established approaches to predict the energy value. Bottom: the methodology developed in this work.

## Methods

Instead of learning the difference between some medium and high quality calculations, we deliberately reduce the basis set quality of the baseline quantum chemistry calculation and, at the same time, add a physical correction, the complementary auxiliary basis set singles (CABSs) correction. Unlike the original idea in F12 methods, we minimize the auxiliary basis, which renders the baseline calculations for our machine learning (ML) models inexpensive, thus significantly reducing the time to result compared with regular Δ-ML approaches.

### Kernel Ridge Regression

For a molecule of given nuclear charges *Z*_*I*_ and positions **R**_*I*_, one can find a representation $x\left(Z_{I},R_{I}\right)\in R^{d}$ for each of *n* molecules $\left(X\in R^{n\times d}\right)$ . With those representations, we employ Kernel-Ridge-Regression (KRR) where a kernel function *k*(*x*, *x*′) acts as a similarity measure in chemical space.

5 $$f\left(x_{q}\right)=\overset{n_{t}}{\underset{i=1}{\sum }}\alpha_{i}k^{G}\left(x_{q},x_{i}\right)$$

where *x*_*q*_ is the representation of the query molecule and the model coefficients α are found by minimizing the loss function

6 $$L=\overset{n_{t}}{\underset{i}{\sum }}{\left(f(x_{i})-y_{i}\right)}^{2}+\lambda {∥f∥}^{2},;,{∥f∥}^{2}\equiv \alpha^{T}K\alpha$$

with the Kernel matrix element **K**_*ij*_ = *k* ^*G*^(*x*_*i*_, *x*_*j*_) and regularization strength λ. The model for a *fixed* representation *x*(*Z*_*I*_, **R**_*I*_) can be obtained directly via

7 $$\alpha ={\left(K+\lambda I\right)}^{-1}y$$

The coefficients **α** implicitly depend on the regularization parameter λ and on the internal parameters of the kernel function *k*, which together are called hyperparameters. To find the optimal combination of hyperparameters **θ** that minimizes the expected value of the loss function, the model in eq 7 is built with different combinations of hyperparameters during cross validation. In this work, we employ nested Monte Carlo cross validation where a full data set *S* is repeatedly partitioned into *p* random disjoint subsets *T*_*i*_ (training) with training size *N* = |*T*_*i*_| and *H*_*i*_ (holdout). For each such split, the training part *T*_*i*_ is further partitioned randomly into *s* shuffles of training data $T_{ij}^{T}$ and the corresponding validation set $T_{ij}^{V}$ , such that $T_{ij}^{V}\cup T_{ij}^{T}=T_{i}$ for all shuffles *j*. Since the loss function eq 6 contains the mean squared error (MSE), we choose the root mean squared error (RMSE) as unit-compatible target metric. We will denote the RMSE for a model trained on data set *A* and predicted on *B* as E(A→B|k,θ) .

For each partition *i*, this yields a validation error

8 $$E\left(T_{i}|k,\theta \right)=\frac{1}{s}\underset{j}{\sum }E\left(T_{ij}^{T}\to T_{ij}^{V}|k,\theta \right)$$

which is minimized to obtain the optimal hyperparameters

9 $$\theta_{i}=\underset{\theta }{\arg\min}\;E\left(T_{i}|k,\theta \right)$$

The expected model accuracy on unseen data (the generalization error) $E_{k}\left(S\right)$ then is

10 $$E_{k}\left(S,N\right)=\frac{1}{p}\underset{i}{\sum }E\left(T_{i}\to H_{i}|k,\theta_{i}\right)$$

This particular method avoids bias from a single particular split and yields a reliable estimate of the underlying generalization error, in particular in the low-data regime, which is of high practical relevance. The random permutations allow for dynamic data set-specific sampling of splits until the results converge w.r.t. the number of permutations *p* and splits *s*. While slightly more expensive than *k*-fold cross validation, it is systematically improvable by keeping the validation set size $\left|T_{ij}^{V}\right|$ independent from the number of splits *s*. We find that the test error closely matches the validation error as expected: $E\left(T_{i}|k,\theta_{i}\right)\approx E\left(T_{i}\to H_{i}|k,\theta_{i}\right)$ .

We speed up and stabilize our hyperparameter optimization using the Schur complement, the eigendecomposition, by using Kernel centering and Chebyshev polynomials (see below).

### Schur Complement

Naive implementation of eq 7 for each model in the cross validation requires either a Cholesky decomposition or a matrix inversion, both $O\left(N^{3}\right)$ . For training, this is the dominant cost, since typically thousands of models are trained to find the correct hyperparameters. While the Cholesky decomposition is numerically more stable, the inversion allows for a particularly efficient implementation where the marginal cost of additional shuffles is much lower (often by 2 orders of magnitude) by using the Schur complement.

First, we calculate the matrix **M**, which is the Kernel matrix of the training and validation data, the size of **M** is (*N*_*q*_ + *N*_*p*_) × (*N*_*q*_ + *N*_*p*_), where *N*_*p*_ is the number of validation points and *N*_*q*_ is the number of training points. This yields the 2 × 2 blockmatrix

11 $$M=\left[\begin{array}{ll} K_{tt} & K_{tv}\\ K_{vt} & K_{vv} \end{array}\right]$$

The submatrices have the size **K**_*tt*_ (*N*_*q*_ × *N*_*q*_), **K**_*tv*_ (*N*_*q*_ × *N*_*p*_), **K**_*vt*_ (*N*_*p*_ × *N*_*q*_) and **K**_*vv*_ (*N*_*p*_ × *N*_*p*_). The inversion of **M** yields another 2 × 2 blockmatrix

12 $$M^{-1}=\left[\begin{array}{ll} E & F\\ G & H \end{array}\right]$$

the sizes of the submatrices will be conserved during the inversion. Since **M** is larger than **K**_*tt*_, this initial inversion comes with a higher cost than the inversion of the matrix **K**_*tt*_. But for further shuffle splits, the matrices **M** and **M** ^–1^ can be shuffled jointly using the permutation matrix **P**

13 $$M^{\prime }=PMP^{T}\Rightarrow M^{\prime -1}=PM^{-1}P^{T}$$

Therefore, it suffices to calculate **M** and **M** ^–1^ once and reuse them for different splits during the cross-validation.

The inversion of a 2 × 2 blockmatrix can be calculated by using the Schur complement, applied on **M** ^–1^, which yields^85^

14 $$K_{tt}={\left(E-FH^{-1}G\right)}^{-1}\Rightarrow K_{tt}^{-1}=E-FH^{-1}G$$

Therefore, the inversion of **K**_*tt*_ can be substituted with the inversion of **H**, which has the size (*N*_*p*_ × *N*_*p*_). With an asymptotic scaling of $O\left(N^{3}\right)$ for matrix inversion and a standard test/train-split of 20/80, the computational cost is reduced to 1/4^3^ ≈ 1.6%, although scaling remains the same.

### Eigendecomposition

The kernel matrix **K** depends only on the parameters of the kernel function for a fixed data set. For this reason, it might be beneficial to calculate the kernel matrix for one set of initial parameters and use the eigendecomposition of **K** to evaluate the performance for different values of the regularization parameter λ. The eigendecomposition of **K** is

15 $$K=Q\Lambda Q^{-1}$$

Here **Q** is the matrix consisting of the eigenvectors of **K** and **Λ** is a diagonal matrix with the eigenvalues λ_*i*_ as its elements. Substituting **K** in eq 7 with eq 15 results in

16 $$Q{\left(\Lambda +\lambda I\right)}^{-1}Q^{T}=QLQ^{T},;,L=\mathrm{diag}\left(\frac{1}{\lambda_{i}+\lambda }\right)$$

Hereby, we substitute a matrix inversion with two matrix multiplications. While the multiplication with a diagonal matrix scales with $O\left(N^{2}\right)$ , the second multiplication scales with $O\left(N^{3}\right)$ and, therefore, has the same formal scaling as a matrix inversion. We apply the eigendecomposition only together with the Schur complement. By inserting eq 14 into eq 7 we obtain

17 $$\alpha =\left(E-{\mathrm{FH}}^{-1}G\right)y=\mathrm{Ey}-\left(F\left(H^{-1}\left(\mathrm{Gy}\right)\right)\right)$$

The submatrices can be expressed by the eigendecomposition of the Matrix **M**

18 $$\begin{array}{ll} E & =Q_{\mathrm{Train}}LQ_{\mathrm{Train}}^{T},;F=Q_{\mathrm{Train}}LQ_{\mathrm{Val}}^{T}\\ H & =Q_{\mathrm{Val}}LQ_{\mathrm{Val}}^{T},;G=Q_{\mathrm{Val}}LQ_{\mathrm{Train}}^{T} \end{array}$$

**Q**_Train_ and **Q**_Val_ are the truncated versions of **Q** from eq 15 that contain only the rows of training points or, respectively, validation points. Using these expressions in eq 17 allows us to write eq 7 as a chain of matrix vector products, which scale with $O\left(N^{2}\right)$ , rather than calculating the matrix inversion. The eigendecomposition of **M** also allows us to skip the inversion of **M**.

### Kernel Centering

The standard approach in KRR is to center the labels by subtracting the mean of the training data. This ensures that the prediction model is not biased by any underlying trend and (in Gaussian Process Regression terminology) aligns the expected prediction with the zero mean function that KRR implies. However, the features of the training data are generally not centered around the origin of the feature space. By using a kernel method like KRR to predict the label of a query molecule as shown in eq 5, the model must map the uncentered representations in the feature space onto the centered labels. To achieve this, the model needs to account for the offset. Therefore, we center our kernel matrix following the expression:^86^

19 $$K^{\prime }=\left(K-1_{N}K-K1_{N}+1_{N}K1_{N}\right)$$

where 1_*N*_ is the matrix with all entries equal to 1/*N*. By centering the kernel matrix, the largest eigenvalue is driven to zero, or numerically close to it, rendering the matrix rank deficient. Although a rank deficient matrix cannot be inverted directly, this is not an issue for KRR, since the inversion is not applied to K alone but to the regularized sum given in eq 7. The eigenvalue zeroed out by centering is therefore shifted by λ, restoring the matrix to full rank. For the data and kernel functions used in this work, the dominant eigenvalue in the uncentered kernel originated from the constant offset. Centering removes this offset, thereby reducing the condition number. A better-conditioned matrix in turn enables the use of decomposition methods such as the Schur complement or eigendecomposition for a larger range of model parameters.

### Chebyshev Polynomials for Local Kernels

For some properties, a local kernel is advantageous where each atom is assigned its own representation $x_{i}^{l}$ or, equivalently, the full molecule is assigned a matrix *x*_*i*_. This forms non-normalized kernel functions *k* ^*U*^ from atomic kernel functions *k* ^*A*^:

20 $$k^{U}\left(x_{i},x_{j}\right)=\overset{N_{A}}{\underset{l=1}{\sum }}\overset{N_{B}}{\underset{m=1}{\sum }}\delta_{Z_{l},Z_{m}}k^{A}\left(x_{i}^{l},x_{j}^{m}\right)$$

with the optional Kronecker-Delta δ, which is 1 if the nuclear charges *Z*_*l*_ and *Z*_*m*_ of the *N*_*A*_ atoms are identical and zero otherwise, which restricts comparison of atomic environments within each element only, suppressing alchemical interactions even if such elemental similarity might exist.^87^ If the Kronecker-Delta is included, we will refer to this as *elemental* kernel, otherwise we will call it a *local* kernel only.

Through normalization, one obtains the local equivalent *k* ^*L*^ to the global kernel *k* ^*G*^ in eq 5:

21 $$k^{L}\left(x_{i},x_{j}\right)=\frac{k^{U}\left(x_{i},x_{j}\right)}{\sqrt{k^{U}\left(x_{i},x_{i}\right)k^{U}\left(x_{j},x_{j}\right)}}$$

During cross-validation, the hyperparameters of the kernel function will be changed iteratively and thereby trigger a full reevaluation of the kernel function on the pairwise distances between all training atoms. The cost of calculating a single kernel matrix element scales quadratically with the average number of atoms in the molecules for a local representation while it is of constant cost for global representations. Since for a radial basis function kernel such as the exponential or the Laplacian kernel, the sum of exponential functions cannot be simplified by just algebraic operations, it would be desirable to have a way of saving at least the unfavorably scaling way of double sum over the pairwise atoms. We obtain constant cost for a single kernel matrix element, even for a local representation with an exponential or Laplacian kernel, by approximating the exponential function over the domain [−20, 0] as Chebyshev polynomial over the domain [−1, 1]. For *x*  > 20, exp­(−*x*) is essentially 0. We reach a maximum absolute error to the exponential over the domain of about 10^–9^ with a polynomial of degree *d* = 20. Approximating $\exp\left(x\right)\approx \overset{d}{\underset{p=0}{\sum }}c_{p}x^{p}$ , we can write e.g., the local exponential kernel in eq 20 as

22 $$k^{U}\left(x_{i},x_{j}\right)=\overset{N_{A}}{\underset{l=1}{\sum }}\overset{N_{B}}{\underset{m=1}{\sum }}\delta_{Z_{l},Z_{m}}\exp\left(-∥x_{i}^{l}-x_{j}^{m}∥/\sigma \right)$$

23 $$\approx \overset{N_{A}}{\underset{l=1}{\sum }}\overset{N_{B}}{\underset{m=1}{\sum }}\delta_{Z_{l},Z_{m}}\overset{d}{\underset{p=0}{\sum }}c_{p}{[\frac{-∥x_{i}^{l}-x_{j}^{m}∥}{\sigma }]}^{p}$$

24 $$=\overset{d}{\underset{p=0}{\sum }}\frac{c_{p}}{\sigma^{p}}\overset{N_{A}}{\underset{l=1}{\sum }}\overset{N_{B}}{\underset{m=1}{\sum }}\delta_{Z_{l},Z_{m}}{\left(-∥x_{i}^{l}-x_{j}^{m}∥\right)}^{p}$$

25 $$=\overset{d}{\underset{p=0}{\sum }}\frac{c_{p}}{\sigma^{p}}C_{ijp}$$

with the kernel function length scale σ. This way, the double sum over the pairwise combinations of atoms can be replaced with a precomputed third-order tensor *C*_*ijp*_ and the evaluation of a polynomial, which can be done in constant time and memory regardless of the number of atoms. The same approximation can be made for the Gaussian kernel by inserting the squared distance and length scale.

### Complementary Auxiliary Basis Set Optimization

We expect the CABSs correction to have diminishing returns with increasing CABS basis size. Therefore, we choose comparably small CABS bases and optimize the exponents to minimize the mean absolute error (MAE). For hydrogen atoms, we always only use S functions. We calculate the gradients of the MAE with forward finite differences and use steepest descent with a line search on a logarithmic grid.

To avoid that CABS basis functions are spent recovering simple known trends, we detrend the BSIE before optimizing the basis. We exploit two such trends: either linear per-element contributions^41,88^ (*atomic* detrending) or additionally nonlinear pair interactions^60^ (*pair* detrending). For atomic detrending

26 $$E_{\mathrm{atomic}}\approx \underset{Z}{\sum }n_{Z}c_{Z}$$

with n_*Z*_ atoms of element *Z*, the coefficients *c*_*Z*_ are obtained via a least-squares fit on the training data. For the pair detrending, we use

27 $$E_{\mathrm{pair}}\approx E_{\mathrm{atomic}}+\underset{i<j}{\sum }Z_{i}^{p}Z_{j}^{p}\exp\left(-\theta R_{ij}\right)$$

with nuclear charges *Z*_*i*_ and interatomic distance *R*_*ij*_. We choose *p* = 2.5 and θ = 3 and keep them fixed throughout this work, since the fit shows a wide parameter range with equivalent results.

To simplify our optimization procedure, we reimplemented the CABSs correction as implemented in the MP2-F12 routines of Psi4 ^39^ in Python using PySCF^89^ (version 2.13.1) and AI assistance and verified the equivalence of the results on 100 molecules (CO, N_2_, water, benzene, rest randomly selected from VQM24 ^90^) where the mean and maximum absolute error in the single correction is 3· 10^–8^ Ha and 5 · 10^–7^ Ha, respectively. For density fitting RHF (benzene) the error is 9 · 10^–5^ Ha. All tests are part of the continuous integration tests of our implementation.

## Results

We selected the GDB-BSIE data set,^48^ which was designed for CBS extrapolation studies, including sequential energy values from increasing cardinal points ranging from STO-3G to cc-pV5Z. Its largest basis set is close to the CBS limit and becomes our learning target. The GDB-BSIE data set contains ten batches of molecular configurations, spanning different conformers. Since we found batch nine thereof to mistakenly report B3LYP total energies in the HF data set, we excluded it from all our considerations.

### CABS Optimization

For our optimization of the CABS basis, we use 200 random molecules from the GDB-BSIE data set. They have been stratified to span the full elemental diversity of the data set: C, H, N, O, F, S, and Cl. Since this selection is particularly small and typically allows only a few parameters per element, we do not expect noticeable overfitting. The timing information has been obtained by comparing the evaluation cost of the Hartree–Fock single point and the additional steps necessary to evaluate the CABS correction using our PySCF implementation.

Figure 2 shows that the key component for an effective and cheap CABS correction over plain pcseg-0 calculations is adding D functions, as almost all points on the Pareto front contain D functions. Since pcseg-0 contains S and P functions only, that matches the common picture that higher cardinal number basis sets introduce basis functions of the corresponding next higher angular momentum. The optimization itself is well-conditioned, since no CABS basis that is a strict superset of a smaller basis set yields a lower error. It is interesting, however, that adding F functions does not improve significantly over D functions alone.

**Figure 2:**
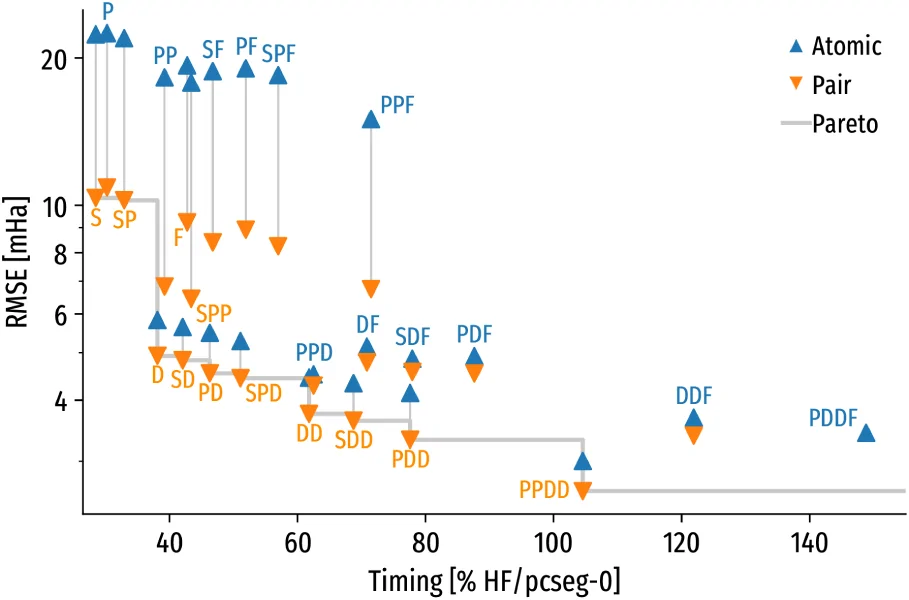
Residual nonlinear error as a function of the computational cost of the optimized CABS correction relative to the HF/pcseg-0 single point for atomic detrending and for pair detrending. The Pareto front connects the points forming the optimal trade-off. The labels denote the different CABS shells for all heavy elements (hydrogen always has one S shell), e.g., PDD denotes a CABS basis of one P and two D shells.

We find that optimizing the CABS exponents without removing the linear trend of atomic contributions is not efficient, suggesting that the CABS correction on its own covers mostly a linear trend in atomic contributions in a comparably costly manner. With detrending, however, CABS applied to the minimal basis pcseg-0 reaches cc-pVDZ accuracy at a fraction of the cost.

We observe that the effect of the pair detrending is strongest if no D functions are included in the CABS and that no pair detrending reaches the accuracy of any form of inclusion of D functions alone, suggesting pairwise contributions to be mostly a subset of D contributions. Pair detrending is particularly effective for small CABS—exactly the case we consider here.

The auxiliary bases in Figure 2 are substantially smaller than generated ones such as AutoCABS, which is essential to our approach. While adding more functions can reduce the CABS error further, Figure 2 clearly shows that this improvement is slow. Higher angular momenta become increasingly costly, since each adds 2*l* + 1 basis functions. We therefore suggest to use a small CABS for CBS estimation in conjunction with atomic or pair detrending and with D functions to save substantial computational cost. In this work, this approach provides a highly efficient baseline for machine learning methods (see below).

### Accelerated Kernel Ridge Regression

Using the combination of kernel centering, Schur complement and eigendecomposition in general, and for local representations Chebyshev polynomials, we achieve a 10×–50× speedup compared to canonical KRR, each on a single core. The vast speed up in training models makes it possible to run cross-validation over a comprehensive grid of hyperparameters.

The models in Figure 3 were trained with a grid of 21 length scales σ and 10 regularization strengths λ with 50 shuffles. The total time *t* needed to build such a model is

28 $$t=t_{D}+n_{\sigma }t_{K}+n_{s}n_{\sigma }n_{\lambda }t_{\alpha }$$

Here *t*_D_ and *t*_K_ are the times to build the pairwise distance and the kernel matrix, while *t*_α_ is the time to solve eq 7, *n*_σ_, *n*_λ_ and *n*_s_ are the number of the corresponding hyperparameter and shuffles used in cross-validation. While this expression is in theory true for both global and local representations, there are some limiting factors. For local representations, the size of the pairwise distance matrix and the kernel matrix are too large for most systems to keep them in memory at the same time, e.g., for 4096 training points, both matrices would each require 53 GB additionally, the local representations would require around 17 GB. Therefore, it is necessary to recalculate both *t*_D_ and *t*_k_ for each σ. Using the approximation of the Chebyshev polynomials for local kernels we can save this costly step.

**Figure 3:**
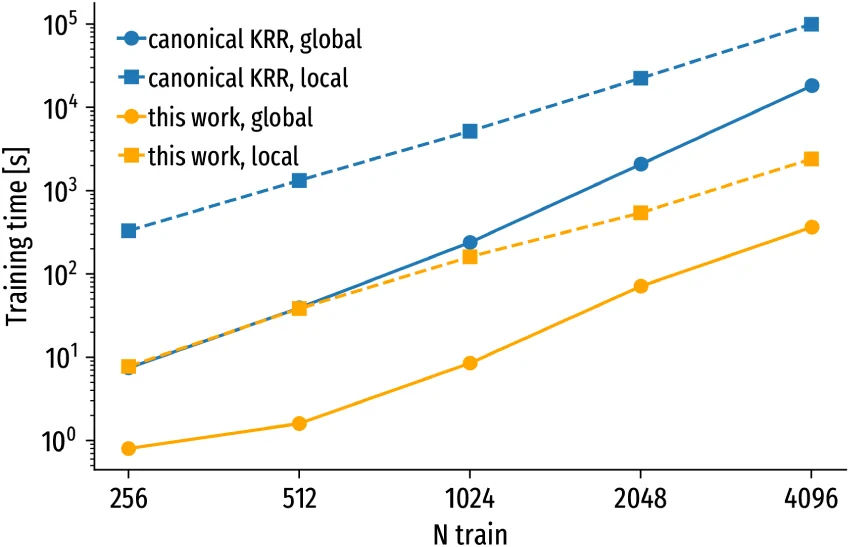
Training time as a function of the training set size. In blue, models trained with a canonical KRR approach; in orange, models trained using the models mentioned above. The models where trained on cc-pV5Z data using the local and global versions of SLATM. All models were trained on an AMD EPYC 7513 using a single core.

By applying all the techniques described in the method part, we can observe a performance increase of more than 1 order of magnitude in comparison to the canonical version of KRR. In the case of the global representation the time for 4096 data points is reduced to 17.5% of the original cost, while the cost for the local version is reduced to 2.4% of the canonical version for the same training set size. However, in this comparison we assumed that we can use the decompositions and the approximation for every pair of hyperparameters on this data set, this is not formally guaranteed for every data set and might lead to different performance curves depending on the data set and hyperparameter grid. Both eigen decomposition and Schur complement have a direct impact on *t*_α_, while the Chebyshev approximation only improves the time *t*_K_ for local representations. In eq 28 we can see that *t* scales with the size of the hyperparameter grid and the number of shuffles, as a result our method becomes more valuable for larger hyperparameter scans.

### Approaching the CBS Limit

#### Two-Point Extrapolation

Extrapolation schemes are the standard way to estimate the CBS limit. While it is commonly known that using the SZ/DZ for the extrapolation yields large errors, we include this approach as an additional illustrative comparison point and assess whether the CABSs correction would improve it, despite the electronic structure calculation with DZ being about nine times more expensive than pcseg-0. This factor formally scales cubically with the number of electrons, so the factor would be even greater for larger molecules. Since published extrapolation parameters are available for DZ and above only, we first needed to obtain the corresponding values for this pair of smaller basis sets. We calculated HF/pcseg-0 for all 131,554 data points in the data set and fitted α based on eq 4.

We obtain α = 5.34 for pcseg-0/DZ extrapolation, as shown in Figure 4. For the remaining cc-pVXZ family pairs DZ/TZ and TZ/QZ, we find α = 4.57 and 6.03, respectively. These values differ slightly from the typical reference values (4.42 and 5.46),^91^ which is expected since the fits are based on different data sets and literature values use uncontracted aug-cc-pV6Z as CBS reference while we use cc-pV5Z.

**Figure 4:**
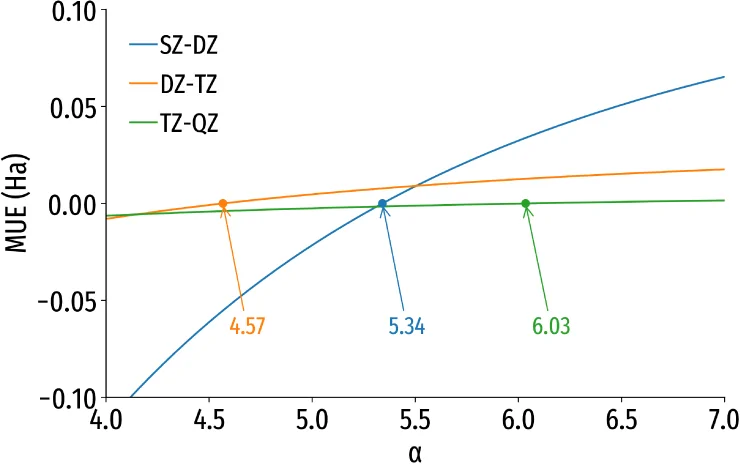
Screening of α values for the extrapolations of SZ/DZ (blue), DZ/TZ (orange), and TZ/QZ (green) vs mean unsigned error (MUE) for 131,554 points.

For our SZ/DZ extrapolation, the residual error of 4.95 mHa is close to that of using only cc-pVDZ (5.47 mHa), both with atomic detrending. This confirms that plain extrapolation from a minimal basis reference is not suitable, since the SZ error is too system-specific, which further motivates our use of the system-specific CABSs correction.

#### Single-Point Calculations

At the heart of the CABSs correction is a basis set which is not used in the Hartree–Fock calculation at all, but comes in a postprocessing step where it is supposed to cover the remaining degrees of freedom that are not described by the original basis set. Besides manual optimization,^40,92^ automated methods have been developed. We use AutoCABS,^93^ which typically has been tested on DZ and larger basis sets so far. Using PSI4 1.10, we retrieve the approximate timing required for that auxiliary basis, and observe that this additional step (dominated by the Fock matrix build) adds up to a total of approximately 60% to the calculation of cc-pVDZ or renders pcseg-0 roughly as expensive as the DZ calculation on its own, and as such adds no direct computational benefit over DZ out-of-the box. The error however is reduced: with pcseg-0 and its generated AutoCABS we achieve 3.89 mHa, better than cc-pVDZ (5.47 mHa), both with atomic detrending.

From our CABS optimization in Figure 2, we include two auxiliary bases: the minimal approach CABS_min_, using only D functions, since their inclusion halved the RMSE, and a slightly larger one (CABS_small_) using PPDD functions, which showed the overall lowest RMSE in Figure 2.

Without detrending, CABS_min_ requires only 5% extra computational cost over pcseg-0, but provides barely a 10% reduction in error. CABS_small_ requires twice the time of a HF/pcseg-0 calculation, but reaches the same error as the comprehensive AutoCABS-generated auxiliary basis, which is three times more expensive. For the purpose of CBS estimation, a minified and optimized CABS basis therefore is more efficient.

With atomic detrending, all three CABS variants reach a low error, ranging from 7.03 mHa for CABS_min_ to 3.89 mHa for the AutoCABS basis. Pair detrending further reduces this to 5.9 mHa and 5.42 mHa for CABS_min_ and CABS_small_ respectively. This is a reflection of the RMSE values observed in Figure 2, where for most cases pair detrending has a lower value than atomic detrending. The results summarized in Table 1 show that detrending applied to the results from any cardinal number yields an RMSE similar to the next cardinal number. Removing the atomic contribution for these high cardinal numbers provides nearly chemical accuracy for cc-pVTZ (1.36 mHa) while successfully reaching chemical accuracy for cc-pVQZ. In this setting, a singles correction from a small CABS basis already captures the dominant contributions, while typically much larger CABS are employed to obtain more accurate energies even at the CABS singles level.^42^ The detrending yields smaller and more homogeneous residuals, which in turn provide an improved target for ML models. It is also cheaper and more accurate for mean-field energies than dual basis or perturbative approaches which consider a DZ or TZ basis as the small baseline to start from.^44,45^

**Table 1:** RMSE against the Target Energy (RHF/cc-pV5Z) in mHa, Shown for (A) Raw Data, (B) Atomic Detrending (in Parentheses: Relative Error Reduction), (C) Δ Machine Learning (in Parentheses: Number of Training Points Required to Reach 1 mHa RMSE), (D) Inference/Query Time Relative to a HF/cc-pVDZ Single Point Calculation

Method	Original	Detrended	Δ-ML (n)	Time
pcseg-0	1149.34	19.62 (98.3%)	1.87 (8192)	11%
CABS_min_/pcseg-0	1007.15	7.03 (99.3%)	1.05 (8192)	16%
CABS_small_/pcseg-0	647.04	5.99 (99.3%)	0.89 (4096)	24%
CABS/pcseg-0	660.21	3.89 (99.4%)	0.89 (1024)	60%
cc-pVDZ	126.29	5.47 (95.7%)	0.95 (512)	100%
Extrapolation/SZ-DZ	9.59	4.95 (48.4%)	0.95 (512)	110%
CABS/cc-pVDZ	49.47	0.94 (98.1%)		160%
cc-pVTZ	29.60	1.36 (95.4%)		1100%
cc-pVQZ	5.94	0.56 (90.6%)		2000%

### Learning the CBS Limit

From a machine learning perspective, the minimal basis energies are a baseline for learning the difference to the CBS limit. This standard Δ-ML approach has already been applied to the GDB-BSIE data set^48^ and (with 129 222 training and validation points using a neural network) achieves a MAE of 1.56 mHa for a SZ baseline and 0.24 mHa for a DZ baseline. All models in this work require quantum chemistry work at inference time that is above the SZ and below the DZ cost. Figure 5 relates the cost of inference time with the raw residual, the detrended ones, and the machine-learning models discussed in the following.

**Figure 5:**
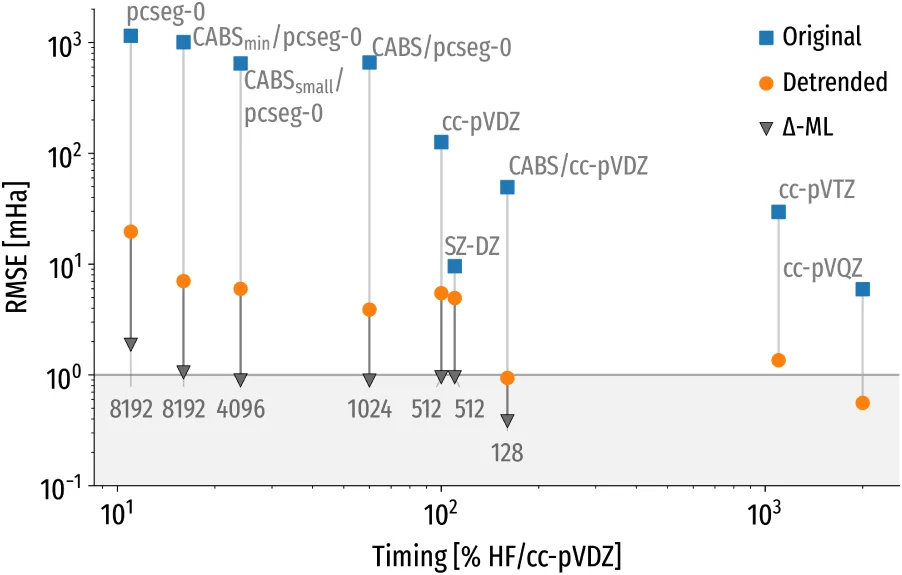
Residual errors as a function of the computational cost of RHF/cc-pVDZ. In blue, the original values; in orange, the values after atomic detrending. The gray points denote the results of a Δ-ML approach with the number of training points as annotations. See Table 1 for details. Target accuracy shaded.

Using our AutoKRR package, we test four different representations: SLATM,^94^ cMBDF,^95^ FCHL19 ^96^ and MACE-MP0 ^97^ (short MACE) for all the methods that would match or decrease state-of-the-art inference cost (a DZ single point), while increasing the accuracy Figure 6. We aim at a target RMSE of 1 mHa.

**Figure 6:**
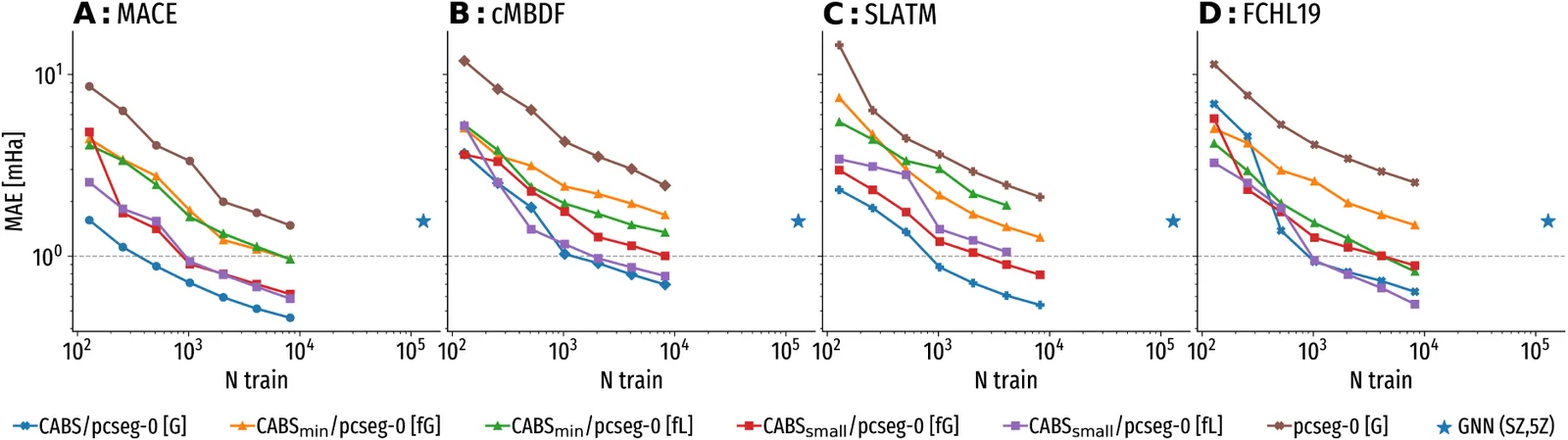
Comparative Δ-ML performance curves for all pcseg-0 approaches using the 4 selected representations studied in this work. Results are shown for (A) MACE, (B) cMBDF, (C) SLATM, and (D) FCHL19, each with local representation [L] or global one [G]. Results from these plots are highlighted in Table 1. Full table in the SI.

We evaluated the global versions of the four representations first, of which MACE was the most data-efficient across all groups. Using it as a representation reaches the target with only 2048 training points with the cheap CABS_small_. In the other extreme, cMBDF required the most training points to reach the target accuracy of 1 mHa. It is the only one that fails to meet the target accuracy for ΔML of RHF-CABS_small_ (within the limit of 8192 evaluated points). SLATM and FCHL19 behave similarly and are between MACE and cMBDF.

Starting from a lower variance should shift the learning curves, making the models more data efficient. This is the motivation behind the detrending approach.

Based on the results of CABS optimization in Figure 2, we learned the residuals of the pair detrending. We find that the pair generally improves the learning curve in the small data regime up to a few hundred data points, but that afterward no noticeable difference between linear and pair detrending is observed, suggesting that the bias induced by the detrending otherwise is learned efficiently by the models.

We also consider local representations with an elemental kernel which are substantially more expensive both in training and at inference time. For an additive quantity, one would expect the local representations to perform better. However, for MACE, there no difference at all between the corresponding learning curves, so using the expensive local kernels does not help. For both cMBDF and FCHL19 representations, the local approach provides better results than the global one, almost reaching the accuracy of the CABS/pcseg-0 model with much higher inference time. For SLATM, local representations perform slightly worse, which may indicate a mismatch between the local kernel structure of SLATM with the structure of the BSIE.

The overall trend in the learning curves supports our approach, exemplified by the particularly clear learning curves for MACE in Figure 6A: the MAE that learning the BSIE with a minimal basis baseline reaches for 8192 points can be achieved with a minimal CABS at 1024 points or our small CABS at 512 points. The AutoCABS basis is even better, reaching the same accuracy at 128 points already, and is still cheaper at inference time than a single DZ calculation. On the same data set, a graph neural network (GNN) requires 129,000 data points to reach the same accuracy^48^ without our improved baseline and detrending.

As shown in the sole figure of the SI, the RMSE of the learning curves tracks the MAE in Figure 6 and is typically 30–40% higher than the MAE, which is expected since the MAE generally bounds the RMSE from below. The tails of the error distribution are closer to a Laplacian (which would yield expected RMSE of 1.41× the MAE) than the one of a Gaussian (at 1.25× the MAE). With the slope of our learning curves, that means that the models reaching an RMSE of 1 mHa require about four times the data to do so than they needed to yield a MAE of that accuracy. As shown in Figure 5, our best models of cost lower than cc-pVDZ achieve the target RMSE with 1.024 and 4.096 data points respectively.

### Gradients

The idea to employ a minimal CABS basis together with a minimal basis set is also applicable to gradients and would either allow particularly cheap geometry optimization (spatial gradients) or accurate quantum alchemy (gradients w.r.t. nuclear charges). Without access to the analytical gradients for CABSs in Molpro,^98^ we differentiate that term numerically. All other gradients (including alchemical ones^99^) are obtained analytically. To realistically benchmark the geometry optimization scenario, we evaluate cc-pVQZ forces on xTB-GFN2 minimum energy configurations of molecules in the GDB-BSIE database. These geometries are realistic starting points of a HF geometry optimization. We also evaluate alchemical gradients on the GDB-BSIE geometries directly. We do not interpolate the CABS exponents between elements as simplifying approximation, essentially assuming that the gradients are already at CBS quality. In both cases, we find that our approach on average yields an accuracy close to a TZ basis. This is in line with earlier work,^98^ which found CABSs corrected DZ calculations to land close to TZ minimum geometries. The data in Table 2 show that the force accuracy improves already with a small CABS which also enables cheaper quantum alchemy expansions, that otherwise require large or specialized bases.^77,78^

**Table 2:** Mean and Maximum Absolute BSIE Errors (MAE and MAXE, Respectively) for Spatial and Alchemical Gradients for Over 500 Molecules from GDB-BSIE^a^

	Spatial gradient		Alchemical gradient	
Method	MAE	MAXE	MAE	MAXE
pcseg-0	8.7 × 10^–3^	4.4 × 10^–2^	5.5 × 10^–2^	1.2 × 10^–1^
cc-pVDZ	3.4 × 10^–3^	1.1 × 10^–2^	7.3 × 10^–3^	1.5 × 10^–2^
CABS_small_/pcseg-0	1.9 × 10^–3^	8.9 × 10^–3^	2.0 × 10^–3^	3.4 × 10^–2^
cc-pVTZ	3.6 × 10^–4^	1.7 × 10^–3^	1.4 × 10^–3^	6.6 × 10^–3^

a All data shown in atomic units

## Conclusions

In this work, we showed that Hartree–Fock energies in the complete basis set limit can be calculated quickly and efficiently by combining minified quantum-chemistry motivated corrections with data-driven learning, rather than classic extrapolation approaches or expensive basis sets. We showed that the BSIE variance can be heavily reduced by simple postprocessing strategies, such as CABS and, especially, atomic detrending, making the residuals smaller and easier to learn. This indicates that the BSIE has a systematic structure that can be captured before applying a machine learning approach. We showed that using inexpensive HF/pcseg-0 calculations augmented with optimized physical corrections and Δ-learning with only 1024 training points, we can recover near-CBS accuracy at a small fraction of the usual computational cost (24% of a DZ calculation). In particular, we found that different CABS variants, such as CABS_small_/pcseg-0, provide a highly favorable cost-accuracy trade-off compared to established approaches. The spatial and alchemical gradients obtained with our minified CABS approach are more accurate than DZ gradients, close to TZ quality. We show that standard Kernel-Ridge-Regression approaches can be accelerated by almost 2 orders of magnitude using Chebyshev polynomials, the eigendecomposition, and the Schur complement. Our implementation, AutoKRR, openly implements these methods and performs full cross-validation and hyperparameter optimization of a model with 1000 training points in a few seconds (global kernel) or two minutes (local kernel) on a single core with modest memory requirements, which makes the methods substantially easier to apply. The results are relevant for those quantum chemistry calculations where the CBS limit of Hartree–Fock needs to be estimated, e.g., for quantum alchemy. Overall, we demonstrate a practical, inexpensive, and scalable CBS estimation approach.

## Supplementary Material



## Data Availability

Our custom implementation of CABS is available in the Python package^100^ nablachem.cabs; all code regarding Kernel Ridge Regression is available as the Python package nablachem.krr. All calculated energies and learning results are available online.^101^

## Notes

Analysis of the root-mean-squared errors of all models, raw data for all learning curves, including validation errors (PDF)
