# Supplementary material for: Data-Driven Complete Basis Set Limit Estimates from a Minimal Auxiliary Basis
Source: J Chem Theory Comput. 2026 Oct 19;22(21):11277–86. doi: 10.1021/acs.jctc.6c01004 (PMC13757760; doi:10.1021/acs.jctc.6c01004)
Supplement: Supplementary file 1 [file ct6c01004_si_001.pdf]

Supporting Information for "Data-driven complete basis set limit estimates from a minimal auxiliary basis"

Nicolas Grimblat[1,2], Gabriel Klassen[1,2], and Guido Falk von Rudorff*[1,2]

[1]Institut für Chemie, Universität Kassel, Heinrich-Plett-Straße 40, 34132 Kassel, Germany
[2]Center for Interdisciplinary Nanostructure Science and Technology (CINSaT), Heinrich-Plett-Straße 40, 34132 Kassel

*Email: vonrudorff@uni-kassel.de

The SI contains information about each model performance for the MAE (main text) and the RMSE in Fig. S1. The raw data is shown as separate tables for CABS/pcseg-0 (Tab. S1), atom-detrended $CABS_{min}$/pcseg-0 (Tab. S2), atom-detrended $CABS_{small}$/pcseg-0 (Tab. S3), pair-detrended $CABS_{min}$/pcseg-0 (Tab. S4), pair-detrended $CABS_{small}$/pcseg-0 (Tab. S5), and pcseg-0 (Tab. S6). These data points are the raw input visualised in the aforementioned figures which are discussed in the main text.

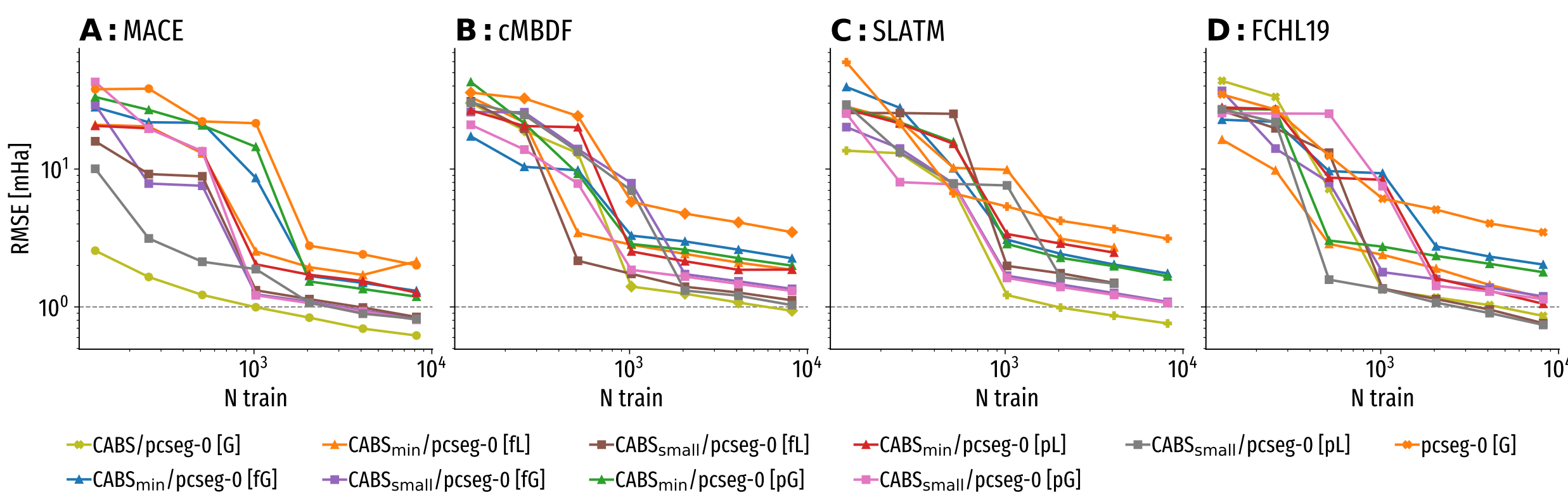

Figure S1: Learning curves, same as Fig. 6 in the main article, just showing the RMSE.

Table S1: Error metrics in mHa as function of training data points for learning CABS/pcseg-0.

Representation	N_{train}	Validation RMSE	Test RMSE	Validation MAE	Test MAE
FCHL19, Global	1	3.869338386	3.891280626	2.787390425	2.795284213
FCHL19, Global	128	1.582452479	43.61392208	1.227304079	6.886992422
FCHL19, Global	256	1.415260682	33.33090132	1.12541206	4.563327412
FCHL19, Global	512	1.342450804	7.15316986	1.048387432	1.383090411
FCHL19, Global	1024	1.239425682	1.334985578	0.939504094	0.931856155
FCHL19, Global	2048	1.085205099	1.170888998	0.818099583	0.818069604
FCHL19, Global	4096	0.92645294	1.030578692	0.710875948	0.730694266
FCHL19, Global	8192	0.794793364	0.860972013	0.611427098	0.637025764
MACE, Global	1	3.992999978	3.887855378	2.851982189	2.815492379
MACE, Global	128	2.045628116	2.55897063	1.530681773	1.577716242
MACE, Global	256	1.58347744	1.64889984	1.203133811	1.121226738
MACE, Global	512	1.041262975	1.222362328	0.813920025	0.880151523
MACE, Global	1024	0.890972087	0.995322743	0.691921173	0.713458446
MACE, Global	2048	0.731134041	0.836155839	0.560285218	0.593479295
MACE, Global	4096	0.634608754	0.695953862	0.483277722	0.513933936
MACE, Global	8192	0.587750572	0.620570836	0.45493698	0.458084102
SLATM, Global	1	3.896382356	3.8881902	2.796693781	2.788323345
SLATM, Global	128	1.790091589	13.58677044	1.421756678	2.319629022
SLATM, Global	256	1.48937154	13.01032342	1.195294609	1.841805774
SLATM, Global	512	1.329052347	7.197611526	1.023657074	1.357036217
SLATM, Global	1024	1.149426807	1.219597511	0.877330229	0.869528778
SLATM, Global	2048	0.918839465	0.989108795	0.700150268	0.711725048
SLATM, Global	4096	0.768349212	0.863807332	0.591509942	0.606860813
SLATM, Global	8192	0.676147431	0.758763778	0.517750323	0.538737755
cMBDF, Global	1	3.874128031	3.88810354	2.795381599	2.787759761
cMBDF, Global	128	1.909862504	30.07875775	1.533180193	3.664994547
cMBDF, Global	256	1.832088359	19.05769107	1.452841121	2.525786378
cMBDF, Global	512	1.615468366	13.02026427	1.249050489	1.855362168
cMBDF, Global	1024	1.415224769	1.404861496	1.076484678	1.028261506
cMBDF, Global	2048	1.181736001	1.247575761	0.887335795	0.913954448
cMBDF, Global	4096	1.015910002	1.075839483	0.773911207	0.792563508
cMBDF, Global	8192	0.870256863	0.937549436	0.67700433	0.697560633

Table S2: Error metrics in mHa as function of training data points for learning linear detrended CABS_{min}/pcseg-0.

Representation	N_{train}	Validation RMSE	Test RMSE	Validation MAE	Test MAE
FCHL19, Global	1	7.073221785	7.06964581	5.126407549	5.1179803
FCHL19, Global	128	4.112383958	22.7845731	3.361909337	5.028563518
FCHL19, Global	256	4.080887097	22.0107581	3.174213526	4.186422441
FCHL19, Global	512	3.530712372	9.658287047	2.713895534	2.967549989
FCHL19, Global	1024	3.046712645	9.318998755	2.254007012	2.588299715
FCHL19, Global	2048	2.793932707	2.74829925	2.029073672	1.961970022
FCHL19, Global	4096	2.418322799	2.317455962	1.771158746	1.69114527
FCHL19, Global	8192	2.056148406	2.028574339	1.521843757	1.483624731
FCHL19, Local	1	7.045006184	7.097609386	5.090624886	5.145877653
FCHL19, Local	128	3.573100799	16.32728638	2.792398897	4.175936948
FCHL19, Local	256	3.172553342	9.78343373	2.47451698	2.948612789
FCHL19, Local	512	2.589542321	2.861412226	1.962542976	1.954771502

FCHL19, Local	1024	2.156735813	2.390159079	1.592751453	1.525309122
FCHL19, Local	2048	1.715552898	1.899577391	1.273235333	1.24994633
FCHL19, Local	4096	1.451012885	1.443538148	1.063279563	1.002823923
FCHL19, Local	8192	1.199505246	1.167460921	0.875249231	0.824064779
MACE, Global	1	7.026476382	7.075791967	5.094143641	5.134260518
MACE, Global	128	3.256019103	28.08894359	2.611243268	4.422908459
MACE, Global	256	2.666284634	21.82887781	2.123771911	3.402101398
MACE, Global	512	2.287732446	21.62749042	1.723308835	2.75889386
MACE, Global	1024	1.876295936	8.617021843	1.414805016	1.783997345
MACE, Global	2048	1.635842669	1.677102512	1.245102984	1.227569853
MACE, Global	4096	1.466920937	1.494000928	1.099542092	1.093659648
MACE, Global	8192	1.325682216	1.308520358	0.995557907	0.965927096
MACE, Local	1	7.085951791	7.076888406	5.166056432	5.150783324
MACE, Local	128	3.103930055	20.93345553	2.456945718	4.083903057
MACE, Local	256	2.785170021	20.37356433	2.166263378	3.354956585
MACE, Local	512	2.58995826	12.96978121	1.984127155	2.471233361
MACE, Local	1024	2.233397064	2.52925896	1.624162672	1.644794943
MACE, Local	2048	1.924161743	1.941901205	1.379938085	1.331045218
MACE, Local	4096	1.648308493	1.706005514	1.176311176	1.129055825
MACE, Local	8192	1.371654132	2.142047784	0.979739398	0.961466346
SLATM, Global	1	7.073831391	7.071855975	5.129157059	5.125763553
SLATM, Global	128	4.186745353	39.34874471	3.369831895	7.447591481
SLATM, Global	256	3.58233999	27.75197592	2.910589802	4.692238825
SLATM, Global	512	3.273244047	10.12119686	2.608906952	3.001194083
SLATM, Global	1024	2.890536818	3.078533829	2.193429372	2.169690342
SLATM, Global	2048	2.451971376	2.427745644	1.810774332	1.700509534
SLATM, Global	4096	2.053317675	2.030407562	1.508905267	1.449868338
SLATM, Global	8192	1.764668107	1.749800294	1.300697705	1.268752642
SLATM, Local	1	6.902971169	7.123888459	5.047725312	5.118893528
SLATM, Local	128	4.118261162	28.40043926	3.159749921	5.476986763
SLATM, Local	256	3.768443993	22.34470836	2.920652335	4.387900957
SLATM, Local	512	3.620922296	10.20323917	2.763319354	3.349407707
SLATM, Local	1024	3.414837173	9.875706293	2.588884424	3.026432431
SLATM, Local	2048	2.966274007	3.126644116	2.211036377	2.206342691
SLATM, Local	4096	2.56256507	2.701166893	1.897003821	1.904220382
cMBDF, Local	1	7.084660856	7.071568914	5.156405828	5.12531065
cMBDF, Global	128	5.041654421	17.24648093	4.023661577	5.072197957
cMBDF, Global	256	4.661349961	10.37811329	3.642624793	3.570999558
cMBDF, Global	512	3.822436044	9.795495009	2.958783364	3.145522094
cMBDF, Global	1024	3.320159208	3.288309275	2.482204516	2.42001309
cMBDF, Global	2048	2.921826365	2.985945706	2.211125934	2.201447561
cMBDF, Global	4096	2.665958147	2.601762426	1.986112735	1.946002285
cMBDF, Global	8192	2.30493819	2.250791467	1.744750244	1.68534791
cMBDF, Global	1	7.099419458	7.037955396	5.155738075	5.128656208
cMBDF, Local	128	3.701454167	33.21899528	3.004077494	5.29125623
cMBDF, Local	256	3.555487951	21.8391531	2.745138826	3.831475043
cMBDF, Local	512	3.250630081	3.444555996	2.414749125	2.408088758
cMBDF, Local	1024	2.643200536	2.825285837	1.965665765	1.949176939
cMBDF, Local	2048	2.282644654	2.423413122	1.685544151	1.709546769
cMBDF, Local	4096	2.075548032	2.095170913	1.507512902	1.488019583
cMBDF, Local	8192	1.867464671	1.861940642	1.381381573	1.351979235

Table S3: Error metrics in mHa as function of training data points for learning linearly detrended $CABS_{small}$/pcseg-0.

Representation	N_{train}	Validation RMSE	Test RMSE	Validation MAE	Test MAE
FCHL19, Global	1	3.928668303	3.931599878	2.792125965	2.788362897
FCHL19, Global	128	2.356524975	36.85752422	1.871957492	5.717817575
FCHL19, Global	256	2.13748797	14.06999672	1.664825617	2.326120259
FCHL19, Global	512	1.868304101	7.993563691	1.42147491	1.753102209
FCHL19, Global	1024	1.639373034	1.786550815	1.24645959	1.265405418
FCHL19, Global	2048	1.522814605	1.589071125	1.139088057	1.118227601
FCHL19, Global	4096	1.371205802	1.380067831	1.03010704	1.005714228
FCHL19, Global	8192	1.216669704	1.192558571	0.911337219	0.8878097
FCHL19, Local	1	3.926087968	3.945977215	2.783971731	2.786349708
FCHL19, Local	128	2.024997227	26.46801939	1.601529596	3.266527758
FCHL19, Local	256	1.827843565	19.73014512	1.476836526	2.530261356
FCHL19, Local	512	1.550226189	13.13145083	1.186994571	1.842358413
FCHL19, Local	1024	1.30510724	1.360622586	0.995214881	0.94774718
FCHL19, Local	2048	1.123899842	1.13978451	0.824858584	0.792000693
FCHL19, Local	4096	0.958079819	0.956830879	0.703879208	0.668285812
FCHL19, Local	8192	0.778760117	0.761687587	0.569092976	0.543951214
MACE, Global	1	3.989815102	3.930869598	2.803176509	2.790972302
MACE, Global	128	1.830589886	29.22811793	1.474070197	4.82379584
MACE, Global	256	1.793112091	7.847944333	1.399528306	1.727223049
MACE, Global	512	1.427325765	7.559292568	1.068348886	1.415483583
MACE, Global	1024	1.225752673	1.234917208	0.924338531	0.904602054
MACE, Global	2048	1.079872426	1.091431329	0.815620726	0.799283033
MACE, Global	4096	0.957700101	0.943935066	0.720192584	0.701523879
MACE, Global	8192	0.847650563	0.827930264	0.635443331	0.618119132
MACE, Local	1	3.940982277	3.873930173	2.78587526	2.764817382
MACE, Local	128	2.02280218	15.8969608	1.608512459	2.551569397
MACE, Local	256	1.707081443	9.194173959	1.310543684	1.820716957
MACE, Local	512	1.455709259	8.849622531	1.099561063	1.558267245
MACE, Local	1024	1.28772673	1.319965475	0.960882052	0.935997242
MACE, Local	2048	1.132000897	1.138545299	0.819557578	0.790317705
MACE, Local	4096	0.94972814	0.989838301	0.689291469	0.677751197
MACE, Local	8192	0.818152782	0.845319742	0.597452014	0.583527049
SLATM, Global	1	3.946340732	3.935066332	2.798690324	2.792942864
SLATM, Global	128	2.123097334	20.12649645	1.717714142	2.976005779
SLATM, Global	256	1.887081159	14.07282621	1.500362059	2.31898992
SLATM, Global	512	1.812166574	7.865012609	1.418331095	1.749245833
SLATM, Global	1024	1.578256044	1.683080198	1.208127526	1.204093219
SLATM, Global	2048	1.436353257	1.45680779	1.073311601	1.046817492
SLATM, Global	4096	1.258043421	1.259782033	0.917517469	0.898777401
SLATM, Global	8192	1.109696454	1.087228184	0.815636229	0.78983416
SLATM, Local	1	3.885610551	3.953983492	2.759141912	2.778791724
SLATM, Local	128	2.143263127	25.47483453	1.733583213	3.421983769
SLATM, Local	256	2.073758035	25.472646	1.623610655	3.114074058
SLATM, Local	512	1.933972084	25.12188346	1.499079385	2.806201758
SLATM, Local	1024	1.847262468	1.984727421	1.41208926	1.408088572
SLATM, Local	2048	1.69095578	1.754221223	1.24712274	1.221934448
SLATM, Local	4096	1.520961911	1.493486374	1.092868042	1.055361145
cMBDF, Local	1	3.905407744	3.933404942	2.75614644	2.780266903
cMBDF, Global	128	2.572592255	25.91652923	2.029700574	3.622476369
cMBDF, Global	256	2.504716498	25.80678464	1.916136736	3.315632357

cMBDF, Global	512	2.196121195	13.96147397	1.662626941	2.273421573
cMBDF, Global	1024	1.912258937	7.886988848	1.454402519	1.759702569
cMBDF, Global	2048	1.720459012	1.727895822	1.304661921	1.274126613
cMBDF, Global	4096	1.546448874	1.53378415	1.176078656	1.141815913
cMBDF, Global	8192	1.37737508	1.352213627	1.035420713	1.00415478
cMBDF, Global	1	3.901330316	3.900673243	2.762910089	2.763847671
cMBDF, Local	128	2.017129395	30.96888581	1.629877006	5.224483684
cMBDF, Local	256	1.930919013	19.62122218	1.490553114	2.547797979
cMBDF, Local	512	1.836926851	2.161412767	1.386069285	1.406556346
cMBDF, Local	1024	1.491319255	1.73730908	1.143188492	1.165337278
cMBDF, Local	2048	1.367509352	1.400732307	1.002515016	0.972317203
cMBDF, Local	4096	1.202431383	1.270655821	0.882368831	0.867642873
cMBDF, Local	8192	1.086945133	1.117177433	0.790573329	0.77763757

Table S4: Error metrics in mHa as function of training data points for learning pair-detrended CABS_{min}/pcseg-0.

Representation	N_{train}	Validation RMSE	Test RMSE	Validation MAE	Test MAE
FCHL19, Global	1	5.860420258	5.881573715	4.599396758	4.624360662
FCHL19, Global	128	3.34084774	27.18734382	2.682187883	4.49311132
FCHL19, Global	256	3.166175434	27.02576536	2.520499182	4.034477256
FCHL19, Global	512	2.875210214	3.030230902	2.255446036	2.225526155
FCHL19, Global	1024	2.55580702	2.735623001	1.951981209	1.965629015
FCHL19, Global	2048	2.326674324	2.344562866	1.749735203	1.728770549
FCHL19, Global	4096	2.100043294	2.054436602	1.57872507	1.51251408
FCHL19, Global	8192	1.817198726	1.784772171	1.362919701	1.324159638
FCHL19, Local	1	5.834478575	5.911635066	4.599871977	4.640230633
FCHL19, Local	128	3.283244905	27.95476997	2.616616869	4.764306668
FCHL19, Local	256	2.928713734	27.21771799	2.221470207	3.566812697
FCHL19, Local	512	2.363673231	8.659253737	1.779523434	2.073488695
FCHL19, Local	1024	1.957597617	8.356087012	1.485111351	1.799867235
FCHL19, Local	2048	1.617441463	1.612334902	1.191613217	1.146908441
FCHL19, Local	4096	1.364945256	1.308041658	0.987721147	0.94165074
FCHL19, Local	8192	1.093320076	1.053505426	0.810125667	0.769591247
MACE, Global	1	5.909375555	5.87823343	4.654539523	4.624958506
MACE, Global	128	2.784441097	33.29572243	2.225878849	4.219856898
MACE, Global	256	2.340146854	26.84877856	1.804319907	3.251450603
MACE, Global	512	2.057499104	20.73188205	1.581041051	2.596339708
MACE, Global	1024	1.717084611	14.48272498	1.319447488	2.024123813
MACE, Global	2048	1.510503661	1.530358692	1.147310874	1.106304142
MACE, Global	4096	1.372609918	1.349009983	1.022959316	0.990221806
MACE, Global	8192	1.219287558	1.186465184	0.909994459	0.881303983
MACE, Local	1	5.873404749	5.896931723	4.614292652	4.639915432
MACE, Local	128	2.814235993	20.56586412	2.211311513	3.853711822
MACE, Local	256	2.678061623	19.72895739	2.067343845	3.010560976
MACE, Local	512	2.335971878	13.42612174	1.729744295	2.359102513
MACE, Local	1024	2.034366356	2.03757071	1.506400354	1.466646192
MACE, Local	2048	1.751788635	1.711488129	1.287814388	1.219206434
MACE, Local	4096	1.466725825	1.539980233	1.089726406	1.056554865
MACE, Local	8192	1.241511187	1.269442467	0.91703818	0.885108307
SLATM, Global	1	6.314475795	6.359539713	4.797545686	4.823789122
SLATM, Global	128	3.718295353	27.8372172	2.895708592	4.909278109
SLATM, Global	256	3.495417631	21.80593704	2.712806887	4.01241694

SLATM, Global	512	3.200170209	15.71787332	2.493953572	3.258891994
SLATM, Global	1024	2.767921022	2.860069528	2.121882804	2.017964107
SLATM, Global	2048	2.306861299	2.270741303	1.718768503	1.636007425
SLATM, Global	4096	1.910161545	1.973751369	1.430943515	1.385967137
SLATM, Global	8192	1.693088705	1.665934415	1.245243249	1.205566284
SLATM, Local	1	5.815327675	5.916356529	4.566626237	4.646867394
SLATM, Local	128	4.090710349	27.19270878	3.30873653	4.979400902
SLATM, Local	256	3.851146041	21.39069104	2.98478768	4.302966781
SLATM, Local	512	3.592697822	15.24233138	2.821382344	3.497327312
SLATM, Local	1024	3.330633406	3.379684111	2.548355381	2.502685559
SLATM, Local	2048	2.931766067	2.878985354	2.16424891	2.094578509
SLATM, Local	4096	2.498103019	2.475645264	1.809824028	1.760769904
cMBDF, Local	1	5.881258314	5.876650357	4.625313235	4.621230566
cMBDF, Global	128	4.20320784	42.79033884	3.417195825	7.780656195
cMBDF, Global	256	3.710164763	21.43313805	2.887724142	3.907624956
cMBDF, Global	512	3.219223916	9.237748145	2.519381095	2.821412435
cMBDF, Global	1024	2.741370435	2.859609996	2.127456621	2.121520355
cMBDF, Global	2048	2.588811045	2.600902021	1.967283127	1.946833506
cMBDF, Global	4096	2.295766188	2.258025815	1.746738147	1.711749981
cMBDF, Global	8192	2.05473294	1.992553145	1.557571706	1.508349148
cMBDF, Global	1	5.88036246	5.876707804	4.633202466	4.62318545
cMBDF, Local	128	3.363866933	26.65092367	2.638219433	4.260260409
cMBDF, Local	256	2.892501495	20.49323116	2.244669343	3.366187995
cMBDF, Local	512	2.579097887	20.09986757	2.001239502	2.979381333
cMBDF, Local	1024	2.329955292	2.525377366	1.754277054	1.746901965
cMBDF, Local	2048	2.05070007	2.144271398	1.525545653	1.491619719
cMBDF, Local	4096	1.799573832	1.858369033	1.325341545	1.29325888
cMBDF, Local	8192	1.59762414	1.866485666	1.188314221	1.18545331

Table S5: Error metrics in mHa as function of training data points for learning pair detrended $CABS_{small}$/pcseg-0.

Representation	N_{train}	Validation RMSE	Test RMSE	Validation MAE	Test MAE
FCHL19, Global	1	3.442850634	3.433168651	2.691151025	2.689754384
FCHL19, Global	128	1.936939253	25.45865743	1.585640913	3.230583227
FCHL19, Global	256	1.836883906	25.24968835	1.472759172	2.818749665
FCHL19, Global	512	1.659722139	25.19528057	1.309723793	2.673744499
FCHL19, Global	1024	1.545116439	7.509492359	1.196754178	1.51818531
FCHL19, Global	2048	1.42402125	1.422741144	1.085038242	1.064888623
FCHL19, Global	4096	1.296618913	1.293841363	0.989564331	0.970112813
FCHL19, Global	8192	1.158318275	1.137107897	0.883972999	0.861934105
FCHL19, Local	1	3.434417396	3.427168408	2.681882609	2.673642667
FCHL19, Local	128	2.010301514	27.09779436	1.59217419	3.280439597
FCHL19, Local	256	1.722386488	21.85458814	1.343406527	2.623582342
FCHL19, Local	512	1.521591077	1.577089576	1.195111518	1.13891385
FCHL19, Local	1024	1.279434012	1.346450437	0.971778922	0.959364532
FCHL19, Local	2048	1.090567606	1.074553072	0.815576076	0.780580652
FCHL19, Local	4096	0.925969992	0.902108196	0.688432501	0.655268762
FCHL19, Local	8192	0.768381032	0.740416085	0.570013889	0.543227224
MACE, Global	1	3.416397108	3.433587481	2.671104514	2.682488058
MACE, Global	128	1.796796691	42.77515502	1.407265485	5.958162788
MACE, Global	256	1.631549978	19.63395514	1.273089548	2.433592811
MACE, Global	512	1.360433271	13.4412894	1.054778766	1.711481977

MACE, Global	1024	1.209823805	1.215003095	0.913526627	0.887662642
MACE, Global	2048	1.073336445	1.06530874	0.819394364	0.781728007
MACE, Global	4096	0.940545613	0.9269489	0.712132557	0.683305492
MACE, Global	8192	0.839126126	0.806542647	0.629916187	0.606110469
MACE, Local	1	3.438733143	3.419526038	2.690575606	2.673248412
MACE, Local	128	1.865511343	10.0418768	1.436476696	2.297721795
MACE, Local	256	1.573243974	3.137126588	1.206657598	1.447624699
MACE, Local	512	1.355603343	2.126446864	1.023199088	1.124173503
MACE, Local	1024	1.202549885	1.88085794	0.899562812	0.955164641
MACE, Local	2048	1.067182742	1.073700431	0.80210598	0.78467406
MACE, Local	4096	0.932975802	0.893457374	0.687903826	0.65750415
MACE, Local	8192	0.800428773	0.818554797	0.597786009	0.578634413
SLATM, Global	1	3.418478315	3.434005349	2.670628305	2.681466814
SLATM, Global	128	2.102808096	25.23835256	1.631330859	4.866823985
SLATM, Global	256	1.967959596	8.032232318	1.561222933	1.887090351
SLATM, Global	512	1.773145223	7.736592018	1.371541715	1.694981192
SLATM, Global	1024	1.613006827	1.623613308	1.22875908	1.201012188
SLATM, Global	2048	1.410054096	1.396648856	1.0604806	1.022556892
SLATM, Global	4096	1.240827032	1.222206183	0.920697069	0.890242821
SLATM, Global	8192	1.089284674	1.067295938	0.806649078	0.778421332
SLATM, Local	1	3.418877675	3.451198467	2.673206616	2.684536842
SLATM, Local	128	2.250978593	29.30805649	1.769839656	3.522244154
SLATM, Local	256	2.07498312	13.23762012	1.649805579	2.387754924
SLATM, Local	512	1.912497176	7.802422117	1.497350825	1.886404649
SLATM, Local	1024	1.83708819	7.608658525	1.399064172	1.723354275
SLATM, Local	2048	1.670653161	1.646051468	1.261211454	1.228484562
SLATM, Local	4096	1.45849699	1.474803529	1.086259298	1.072023312
cMBDF, Local	1	3.421358879	3.433692542	2.67859753	2.690030904
cMBDF, Global	128	2.393298962	20.92601277	1.943613377	3.521352268
cMBDF, Global	256	2.144516442	13.83852268	1.72446127	2.416601615
cMBDF, Global	512	1.935472392	7.850086085	1.502606557	1.883848728
cMBDF, Global	1024	1.796599099	1.856403439	1.381012941	1.378621719
cMBDF, Global	2048	1.646199681	1.659996969	1.259346663	1.2483653
cMBDF, Global	4096	1.482004396	1.466834625	1.124467661	1.113109165
cMBDF, Global	8192	1.330432618	1.306029384	1.01699564	0.995270421
cMBDF, Global	1	3.423655827	3.419128922	2.671594507	2.666869364
cMBDF, Local	128	1.879510135	30.25657124	1.516011579	3.471092414
cMBDF, Local	256	1.810163658	24.69103677	1.417817848	2.806087937
cMBDF, Local	512	1.636205304	13.357053	1.264722045	1.937611776
cMBDF, Local	1024	1.46402153	6.959208864	1.098595926	1.385881191
cMBDF, Local	2048	1.271113489	1.314525597	0.970595748	0.967342057
cMBDF, Local	4096	1.116957033	1.20627956	0.841194003	0.833034424
cMBDF, Local	8192	1.019193014	1.03024764	0.760824623	0.749414539

Table S6: Error metrics in mHa as function of training data points for learning pcseg-0.

Representation	N_{train}	Validation RMSE	Test RMSE	Validation MAE	Test MAE
FCHL19, Global	1	19.49609908	19.64919032	12.31463456	12.47347869
FCHL19, Global	128	10.33022908	34.74147859	8.42951995	11.37099431
FCHL19, Global	256	8.226387518	27.01018462	6.417669972	7.67745639
FCHL19, Global	512	6.785401558	12.46938275	5.134900243	5.298724092
FCHL19, Global	1024	5.71278765	6.097825487	4.250931862	4.105583627

FCHL19, Global	2048	4.416460764	5.077656903	3.410245416	3.441433533
FCHL19, Global	4096	3.74162728	4.0318848	2.849004924	2.925050911
FCHL19, Global	8192	3.257241852	3.477286061	2.50925615	2.543594244
MACE, Global	1	20.33480662	19.62498835	12.81539212	12.54688073
MACE, Global	128	7.263252565	37.90005633	5.503147004	8.598176147
MACE, Global	256	4.84413243	38.23905264	3.723568779	6.310062666
MACE, Global	512	3.92056619	22.13838584	3.006060202	4.070626739
MACE, Global	1024	3.396553189	21.48253834	2.478949984	3.340692724
MACE, Global	2048	2.614395889	2.778477027	1.990812059	1.991509419
MACE, Global	4096	2.197806817	2.406504942	1.677357992	1.731278958
MACE, Global	8192	1.872109594	2.006247375	1.419866019	1.476743958
SLATM, Global	1	19.24768047	19.65125646	12.29636821	12.43627592
SLATM, Global	128	7.177029081	59.48534123	5.742086021	14.52147843
SLATM, Global	256	7.093184397	21.20389435	5.45597916	6.348293158
SLATM, Global	512	5.938620932	6.668457303	4.395675995	4.45060184
SLATM, Global	1024	5.058534433	5.345661972	3.695481234	3.633717788
SLATM, Global	2048	3.92947694	4.217821426	2.902107835	2.923464517
SLATM, Global	4096	3.312695267	3.6700578	2.438923412	2.461256639
SLATM, Global	8192	2.743668713	3.130078858	2.016973333	2.115766475
cMBDF, Global	1	19.1370714	19.66494118	12.2436435	12.41277048
cMBDF, Global	128	10.30529743	35.8288439	8.118959799	11.8842069
cMBDF, Global	256	7.679504079	32.52580937	6.020506439	8.313955195
cMBDF, Global	512	6.267549204	24.22567098	4.855850156	6.381262177
cMBDF, Global	1024	5.396446151	5.788996082	4.163388151	4.271939557
cMBDF, Global	2048	4.543591868	4.751171995	3.492535422	3.529382177
cMBDF, Global	4096	3.705080237	4.10139343	2.881821879	3.020998794
cMBDF, Global	8192	3.124384035	3.483719833	2.404625474	2.44894319
